# Survival outcomes of adjuvant taxanes, platinum plus fluoropyrimidines versus platinum and fluoropyrimidines for gastric cancer patients after D2 gastrectomy: a retrospective propensity score-matched analysis

**DOI:** 10.1186/s12957-021-02390-4

**Published:** 2021-09-10

**Authors:** Lili Wu, Ying Feng, Zhijun Wu, Hui Xu, Cheng Zhang, Jie Ning, Rong Wang, Jianqiong Chen, Minmin Xie, Yi Zhang, Lijia Bu, Jiqing Hao, Tai Ma

**Affiliations:** 1 Department of Oncology, The First Affiliated Hospital of Anhui Medical University, No. 218 Jixi Road, Hefei, Anhui Province 230022 People’s Republic of China; 2 School Clinic, Anhui Medical University, Hefei, Anhui Province 230022 People’s Republic of China; 3 Department of Oncology, Ma’anshan People’s Hospital, Ma’anshan, Anhui Province 243000 People’s Republic of China; 4 Anhui Institute for Cancer Prevention and Control, Hefei, Anhui Province 230022 People’s Republic of China

**Keywords:** Gastric cancer, Adjuvant chemotherapy, Survival analysis, Propensity score matching

## Abstract

**Background:**

To evaluate whether the addition of taxanes to platinum and fluoropyrimidines in adjuvant chemotherapy would result in longer survival than platinum plus fluoropyrimidines in gastric cancer patients who received D2 gastrectomy.

**Methods:**

Data of patients with gastric adenocarcinoma who received D2 gastrectomy and adjuvant chemotherapy with platinum plus fluoropyrimidines or taxanes, platinum plus fluoropyrimidines was retrospectively collected and analyzed. 1:1 Propensity score matching analysis was used to balance baseline characteristics between two groups. Survival curves were estimated using Kaplan-Meier method, and the differences were compared using the log-rank test.

**Results:**

Four hundred twenty-five patients in the platinum plus fluoropyrimidines group and 177 patients in the taxanes, platinum plus fluoropyrimidines group were included into analysis. No statistical differences in disease-free survival and overall survival were observed between two groups. After propensity score matching, 172 couples of patients were matched, the baseline characteristics were balanced. The median disease-free survival were 15.8 months (*95% CI*, 9.3~22.4) in the platinum plus fluoropyrimidines group and 22.6 months (*95% CI*, 15.9~29.4) in the taxanes, platinum plus fluoropyrimidines group (*HR* = 0.63; *95% CI*, 0.48~0.85; *P* = 0.002). The median overall survival was 25.4 months for patients in the platinum plus fluoropyrimidines group (*95% CI*, 19.4~31.3) and 33.8 months (*95% CI*, 23.5~44.2) for those in the taxanes, platinum plus fluoropyrimidines group (*HR* = 0.68; *95% CI*, 0.53-0.87; *log-rank test*, *P* = 0.002).

**Conclusions:**

For gastric adenocarcinoma patients, the adjuvant triplet combination of taxanes, platinum, and fluoropyrimidines regimen after D2 gastrectomy was superior to platinum plus fluoropyrimidines regimen in disease-free survival as well as overall survival.

**Trial registration:**

This project has been registered in the Chinese Clinical Trial Registry (ChiCTR1800019978).

## Introduction

It is estimated that there are more than 1 million incident gastric cancer cases worldwide every year, of which 44% were diagnosed in China [1]. For resectable cases, surgery is the only curative approach, either alone or in combination with perioperative treatment. Radical gastrectomy with D2 lymphadenectomy followed by adjuvant chemotherapy is now considered as standard of care for most gastric cancers in Asian countries [2]. Monotherapy S1 or doublet oxalipatin plus capecitabine regimens were frequently used in adjuvant settings [3–7]. In fact, in actual clinical practice, varied doublet combinations of platinum (oxalipatin, cisplatin, or lobaplatin) with fluoropyrimidines (intravenous 5-fluorouracil, oral capecitabine or S1) were all universally acceptable adjuvant regimens.

Taxanes, which mainly refer to docetaxel and paclitaxel, are effective in gastric cancer treatment and were also tested in adjuvant settings. In the Stomach Cancer Adjuvant Multi-Institutional Group Trial (SAMIT), sequential paclitaxel followed by oral fluoropyrimidines as adjuvant chemotherapy for T4a/b gastric cancer did not improve disease-free survival (DFS) compared to oral fluoropyrimidines [8]. However, recently, docetaxel plus S1 was demonstrated to be superior to S1 monotherapy in stage III gastric cancer treatment [9]. Triplet combination regimens added with taxanes are usually used in perioperative or palliative settings for patients with good performance status [10, 11]. Whether the addition of docetaxel or paclitaxel to the traditional combination of platinum and fluoropyrimidines as adjuvant regimen could improve survival for gastric cancer had not been explored in clinical trials.

By using real-world data from clinical practice, this retrospective study attempted to investigate the DFS as well as overall survival (OS) of the triplet combination regimen with taxanes over doublet regimens in the adjuvant chemotherapy of patients with resectable gastric cancer after D2 gastrectomy, furthermore, subgroup analysis was also plotted to identify which subsets of patients may benefit more from triplet adjuvant chemotherapy.

## Methods

### Patients

This retrospective study consisted of patients diagnosed with gastric adenocarcinoma in the First Affiliated Hospital of Anhui Medical University, Hefei, Anhui Province, China, from 2008 to 2018. All patients included in the study met the following criteria: underwent curative D2 gastrectomy followed by adjuvant chemotherapy; with detailed postoperative pathological report and could be staged according to the American Joint Committee on Cancer (AJCC) seventh staging system [12]; with definite medical records of radiological follow-up after surgical resection; received at least one cycle of systemic adjuvant chemotherapy; adjuvant chemotherapeutic regimens should be either doublet combination of platinum and fluoropyrimidines or triplet combination of taxanes, platinum, and fluoropyrimidines. For platinum drugs, it could be cisplatin, oxaliplatin, or lobaplatin; for fluoropyrimidine compounds, intravenous 5-fluorouracil transfusion, oral capecitabine tablets, or tegafur-gimeracil-oteracil potassium capsules were all acceptable; taxanes could either be docetaxel or paclitaxel. In consideration of tolerance to toxicity, drug accessibility, medical insurance, etc., patients whose adjuvant chemotherapeutic drugs were switched from one platinum drug to another, or one fluoropyrimidine compound to another, or docetaxel to paclitaxel (vice versa) in different cycles were considered eligible. Patients who met any of following criteria were excluded from the study: did not receive curative surgery or not D2 gastrectomy; received adjuvant chemotherapy with other regimens; received neoadjuvant chemotherapy or chemoradiotherapy; treatment was interrupted during the first cycle of chemotherapy; with missing information on surgery, pathology, or adjuvant treatment.

### Data collection and follow-up

We retrieved the medical records of the patients from the Hospital Information System (HIS) and reviewed all related medical files. The following variables were collected: demographic variables including age and sex as well as pathological variables including tumor location, subtype, grade, tumor infiltration (T category), regional lymph node involvement (N category), vascular or perineural invasion by tumors, and perigastric tumor deposits in pathological specimens. Treatment information, including laparotomic approaches, scope of gastrectomy, type of lymphadenectomy, adjuvant chemotherapeutic drugs, date of the first and last dose of chemotherapeutic drug, and cycles of adjuvant chemotherapy were obtained. Staging groups were derived from the T and N categories according to the AJCC seventh staging system. The time to chemotherapy after surgery was defined as interval days from primary surgery to the first dose of adjuvant chemotherapy. The duration of adjuvant chemotherapy was defined as interval days from the first to the last dose of chemotherapeutic drug. Recurrence and metastasis were defined as tumor recurrence in situ (anastomotic stoma or gastric remnant) or metastasis to distant organs, lymph node(s), or intraperitoneal implantation after gastrectomy, with evidence of either radiology, cytology, or histopathology. Vital status was obtained from death records in the death register system or telephonic follow-up to the patients or their relatives. The last date of follow-up was 10 November 2020.

### Propensity score matching analysis

Propensity score matching (PSM) analysis was introduced into our study to balance baseline characteristics between two groups. PSM was performed in SPSS 22.0. This procedure runs a logistic regression on the group indicator and then uses the resulting propensity variable to select controls for the cases. The matching algorithm was the nearest neighbor matching with 1:1 ratio and the caliper was 0.02, the estimation algorithm was logistic regression with “age group,” “sex,” “tumor location,” “Borrmann subtype,” “grade,” “T category,” “N category,” “staging group,” “vascular invasion,” “perineural invasion,” “tumor deposits,” “laparotomic approaches,” and “scope of gastrectomy” as covariates.

### Statistical analysis

DFS was defined as interval months between gastrectomy and first evidence of recurrence or metastasis or last follow-up date (for those with missing data on recurrence or without recurrence). OS was defined as interval months between gastrectomy and death date or last follow-up date. For categorical variables, the Chi-square test was used to examine the differences between the two groups; for numerical variables and nonparametric independent samples, the Mann-Whitney *U* test was used. Survival curves were calculated using the Kaplan-Meier method, and differences were compared using the log-rank test. Hazard ratios (HRs) with 95% confidence intervals (CIs) and two-sided *P* values were reported. HRs in subgroups according to baseline characteristics and two-tailed 95% CIs were calculated using the Cox proportional hazards model. All statistical analyses were performed using SPSS 22.0 (SPSS Inc., Chicago, IL, USA). Two-sided *P* < 0.05 was considered as statistically significant. GraphPad Prism 5.01 software (GraphPad Software Inc., San Diego, CA, USA) was used to draw survival curves and forest maps of the subgroup analysis.

## Results

### Baseline characteristics of the patients

**Fig. 1:**
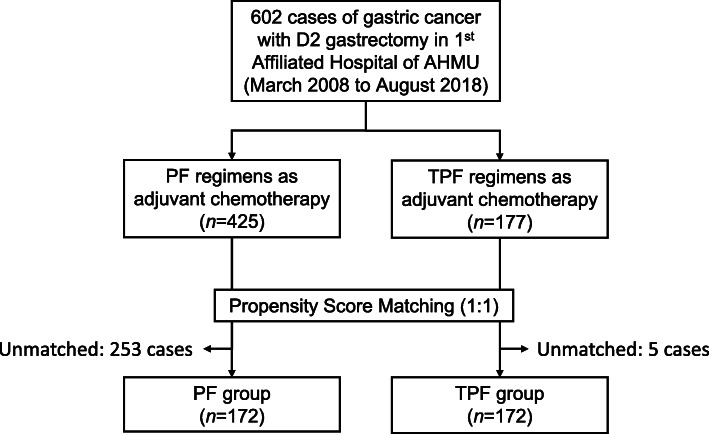
Flowchart of data selection. 1st Affiliated Hospital of AHMU, The First Affiliated Hospital of Anhui Medical University (China); PF, platinum plus fluoropyrimidines; TPF, taxanes, platinum plus fluoropyrimidines

Figure 1 shows the flowchart of case selection. A total of 602 eligible patients were included into analysis; all patients were diagnosed between March 2008 and August 2018. Of them, 425 patients received systemic adjuvant chemotherapy with platinum plus fluoropyrimidines (PF group), and 177 patients received chemotherapy with triplet combination of taxanes, platinum, and fluoropyrimidines (TPF group). By 10 November 2020, total of 332 patients (245 cases in the PF group and 87 cases in the TPF group) developed relapse or metastasis events; 330 and 123 deaths had occurred in the PF and TPF group, respectively. The characteristics between the two groups were compared and results are shown in Table 1. Patients in the TPF group were younger than patients in the PF group (median age, 58 [*IQR*, 48~63] vs. 61 [52~67] years old, *P* < 0.001). The other demographic characteristics, pathological factors, surgical details, and blood parameters (hemoglobin and serum albumin) of the patients were comparable between the PF group and TPF group. After PSM, 172 patients in the TPF group were successfully matched with 172 patients in the PF group (as shown in Table 1), the distribution of age were balanced (median age, 58 [48~63] vs. 61 [51~64] years old, *P* < 0.001), moreover, other variables were still comparable.

**Table 1:** Characteristics of patients in PF group and TPF group before and after propensity score matching analysis

	Before PSM				After PSM			
Variables	PF group [*n* (%), *n* = 425]	TPF group [*n* (%), *n* = 177]	*X*^2^*/Z*	*P*	PF group [*n* (%), *n* = 172]	TPF group [*n* (%), *n* = 172]	*X*^2^*/Z*	*P*
Age (years) [*median* (*IQR*)]	61 (52~67)	58 (48~63)	−4.017^a^	<0.001	60 (51~64)	58 (48~63)	−1.399^a^	0.162
< 65	271 (63.8)	141 (79.7)	14.618	< 0.001	130 (75.6)	136 (79.1)	0.597	0.440
65~	154 (36.2)	36 (20.3)			42 (24.4)	36 (20.9)		
Sex			0.383	0.536			0.234	0.628
Male	313 (73.6)	126 (71.2)			127 (73.8)	123 (71.5)		
Female	112 (26.4)	51 (28.8)			45 (26.2)	49 (28.5)		
Tumor location			2.348	0.125			0.187	0.665
Cardia	214 (50.4)	77 (43.5)			81 (47.1)	77 (44.8)		
Non-cardia	211 (49.6)	100 (56.5)			91 (52.9)	95 (55.2)		
Borrmann subtype			0.760	0.944			2.785	0.594
I	13 (3.1)	5 (2.8)			7 (4.1)	5 (2.9)		
II	125 (29.4)	57 (32.2)			46 (26.7)	57 (33.1)		
III	241 (56.7)	94 (53.1)			101 (58.7)	90 (52.3)		
IV	26 (6.1)	12 (6.8)			12 (7.0)	11 (6.4)		
Unknown	20 (4.7)	9 (5.1)			6 (3.5)	9 (5.2)		
Grade			1.452	0.484			1.356	0.508
G1-2	111 (26.1)	38 (21.5)			41 (23.8)	37 (21.5)		
G3-4	285 (67.2)	126 (71.2)			113 (65.7)	122 (70.9)		
Unknown	29 (6.8)	13 (7.3)			18 (10.5)	13 (7.6)		
T stage (AJCC 7th)			2.315	0.510			0.062	0.996
T1	13 (3.1)	10 (5.6)			10 (5.8)	9 (5.2)		
T2	26 (6.1)	10 (5.6)			10 (5.8)	10 (5.8)		
T3	244 (57.4)	100 (56.5)			97 (56.4)	97 (56.4)		
T4	142 (33.4)	57 (32.2)			55 (32.0)	56 (32.6)		
N stage (AJCC 7th)			4.149	0.246			2.971	0.396
N0	86 (20.2)	25 (14.1)			33 (19.2)	25 (14.5)		
N1	80 (18.8)	42 (23.7)			30 (17.4)	41 (23.8)		
N2	113 (26.6)	50 (28.2)			54 (31.4)	50 (29.1)		
N3	146 (34.4)	60 (33.9)			55 (32.0)	56 (32.6)		
Stage (AJCC 7th)			3.840	0.147			0.528	0.768
I	17 (4.0)	12 (6.8)			11 (6.4)	11 (6.4)		
II	137 (32.2)	46 (26.0)			52 (30.2)	46 (26.7)		
III	271 (63.8)	119 (67.2)			109 (63.4)	115 (66.9)		
Vascular invasion			1.899	0.168			0.134	0.714
Yes	102 (24.0)	52 (29.4)			44 (25.6)	47 (27.3)		
No	323 (76.0)	125 (70.6)			128 (74.4)	125 (72.7)		
Perineural invasion			0.001	0.979			0.091	0.763
Yes	70 (16.5)	29 (16.4)			25 (14.5)	27 (15.7)		
No	355 (83.5)	148 (83.6)			147 (85.5)	145 (84.3)		
Tumor deposits			1.546	0.214			0.595	0.441
Positive	47 (11.1)	26 (14.7)			27 (15.7)	22 (12.8)		
Negative	378 (88.9)	151 (85.3)			145 (84.3)	150 (87.2)		
Laparotomic approaches			0.454	0.500			0.000	1.000
Open	384 (90.4)	163 (92.1)			158 (91.9)	158 (91.9)		
Laporascopy	41 (9.6)	14 (7.9)			14 (8.1)	14 (8.1)		
Scope of gastrectomy			1.451	0.228			0.212	0.645
Total gastrectomy	302 (71.1)	117 (66.1)			118 (68.6)	114 (66.3)		
Partial gastrectomy	123 (28.9)	60 (33.9)			54 (31.4)	58 (33.7)		
Hemoglobin (g/L) [*median* (*IQR*)]	111 (98~121)	115 (102~123)	1.647^a^	0.100	111 (98~120)	114 (102~123)	1.565^a^	0.118
Albumin (g/L) [*median* (*IQR*)]	38.6 (35.6~41.9)	39.3 (36.0~42.7)	1.059^a^	0.290	38.0 (34.3~41.6)	39.2 (36.0~42.6)	1.776^a^	0.076

^a^Independent-samples Mann-Whitney *U* test

*PF*, platinum plus fluoropyrimidines; *TPF*, taxanes, platinum plus fluoropyrimidines; *PSM*, propensity score matching analysis; *IQR*, inter quartile range. *AJCC 7th*, the seventh edition of cancer staging system by the American Joint Committee on Cancer

### Details of adjuvant chemotherapy in the two groups

The details of adjuvant chemotherapy are shown in Table 2. With regards to chemotherapeutic drugs, 27.8% of patients in the PF group were treated with intravenous 5-Fu, 42.8% with oral capecitabine, and 29.4% with tegafur-gimeracil-oteracil potassium, while in TPF group the frequencies of 5-Fu, capecitabine, and tegafur-gimeracil-oteracil potassium administration were 55.4%, 20.9%, and 23.7%, respectively (χ^*2*^ test, *P* < 0.001). For platinum administration, most of the patients (93.6%) in the PF group were treated with oxalipatin, the proportion in TPF group was 49.7%, and there were 45.2% patients in TPF group received cisplatin therapy. In the TPF group, 105 patients (59.3%) were treated with docetaxel and 72 patients (40.7%) were treated with paclitaxel. The median time to chemotherapy after surgery was 38 [*IQR*, 31~47] days in the PF group and 37 [31~47] days in the TPF group (nonparametric test, *P* = 0.867). There was also no difference between the durations of adjuvant chemotherapy in the two groups (median time 116 [53~159] days in the PF group vs. 127 [74~175] days in the TPF group, *P* = 0.081). The finished cycles of adjuvant chemotherapy in the two groups were the same (*P* = 0.477).

**Table 2:** Details of adjuvant chemotherapy in the PF group and TPF group

	PF group [*n* (%), *n* = 425]	TPF group [*n* (%), *n* = 177]	*X*^2^*/Z*	*P*
Taxanes				
Paclitaxel	—	72 (40.7)	—	—
Docetaxel	—	105 (59.3)	—	—
Fluoropyrimidines				
5-Fu	118 (27.8)	98 (55.4)	44.493	< 0.001
Capecitabine	182 (42.8)	37 (20.9)		
S1	125 (29.4)	42 (23.7)		
Platinum				
Cisplatin	20 (4.7)	80 (45.2)	158.765	< 0.001
Oxaliplatin	398 (93.6)	88 (49.7)		
Lobaplatin	7 (1.6)	9 (5.1)		
Time to chemotherapy after surgery (days) [*median* (*IQR*)]	38 (31~47)	37 (31~47)	−0.167^a^	0.867
Duration of adjuvant chemotherapy (days) [*median* (*IQR*)]	116 (53~159)	127 (74~175)	−1.743^a^	0.081
Cycles of adjuvant chemotherapy [*median* (*IQR*)]	4 (2~6)	4 (3~6)	−0.711^a^	0.477

^a^Independent-samples Mann-Whitney *U* test

*PF*, platinum plus fluoropyrimidines; *TPF*, taxanes, platinum plus fluoropyrimidines; *IQR*, inter quartile range

### Disease-free survival

**Fig. 2:**
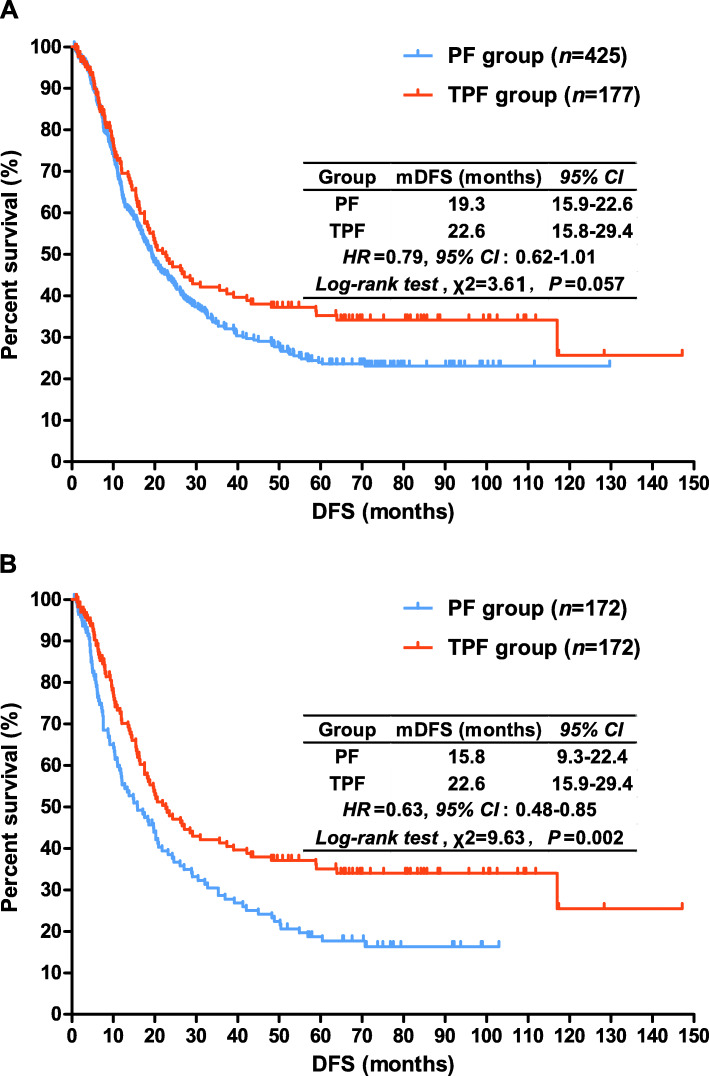
Kaplan-Meier estimated disease-free survival curves for the gastric cancer patients with adjuvant chemotherapy before (**A**) and after (**B**) propensity score matching analysis. PF, platinum plus fluoropyrimidines; TPF, taxanes, platinum plus fluoropyrimidines; mDFS, median disease-free survival; HR, hazard ratio

The median DFS was 19.3 months (*95% CI*, 15.9~22.6) in the PF group and 22.6 months (*95% CI*, 15.8~29.4) in the TPF group (*HR* = 0.79; *95% CI*, 0.62-1.01; log-rank test, *P* = 0.057). The difference was of margin significance; survival curves for DFS were estimated by Kaplan-Meier method and are shown in Fig. 2A. After balancing the baseline characteristics by PSM, the median DFS of 172 matched patients in the PF group was 15.8 months (*95% CI*, 9.3~22.4), while in the TPF group, the median DFS was 22.6 months (*95% CI*, 15.9~29.4) (log-rank test, *P* = 0.002). Addition of taxanes in adjuvant chemotherapy of gastric cancer could decrease risk of relapse by 37% (*HR* = 0.63; *95% CI*, 0.48~0.85). Kaplan-Meier estimated survival curves are depicted in Fig. 2B.

### Overall survival

**Fig. 3:**
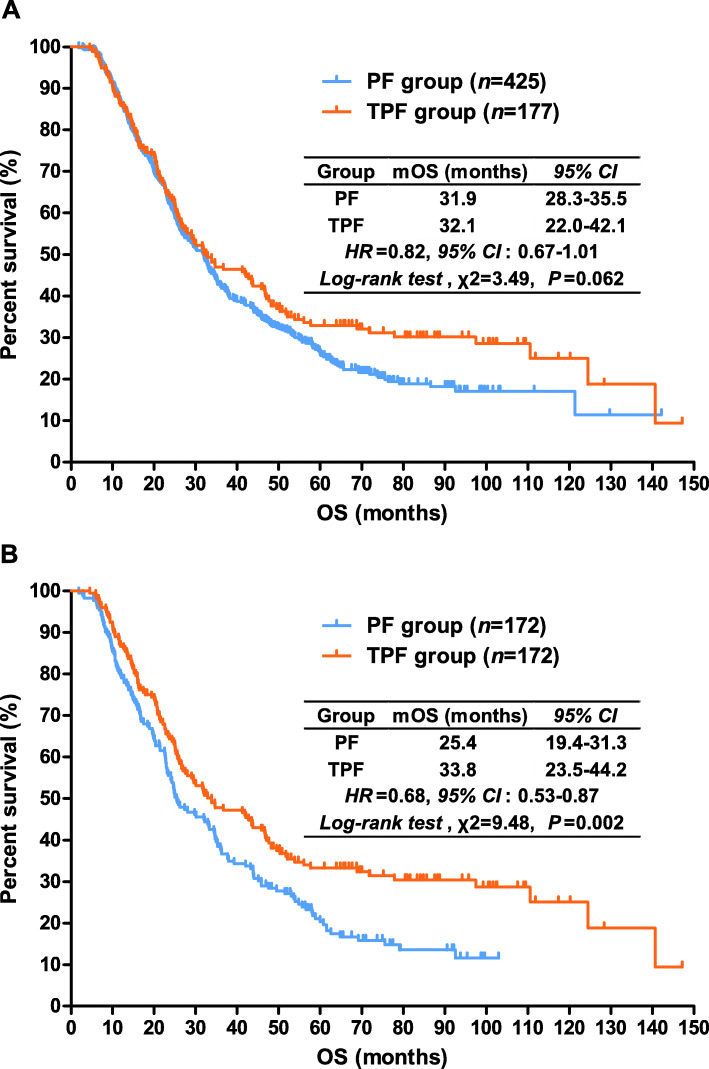
Kaplan-Meier estimated overall survival curves for the gastric cancer patients with adjuvant chemotherapy before (**A**) and after (**B**) propensity score matching analysis. PF, platinum plus fluoropyrimidines; TPF, taxanes, platinum plus fluoropyrimidines; mOS, median overall survival; HR, hazard ratio

As shown in Fig. 3A, the median OS was 31.9 months for patients in the PF group (*95% CI*, 28.3~35.5) and 32.1 months (*95% CI*, 22.0~42.1) in the TPF group (*HR* = 0.82; *95% CI*, 0.67-1.01; log-rank test, *P* = 0.062). Figure 3B showed survival curves for OS after PSM, the median OS for patients in TPF group was significantly longer than those in PF group (33.8 vs. 25.4 months, *P* = 0.002). Mortality risk was significantly decreased in patients who received triplet adjuvant chemotherapy (*HR* = 0.68; *95% CI*, 0.53~0.87).

### Subgroup analysis

**Fig. 4:**
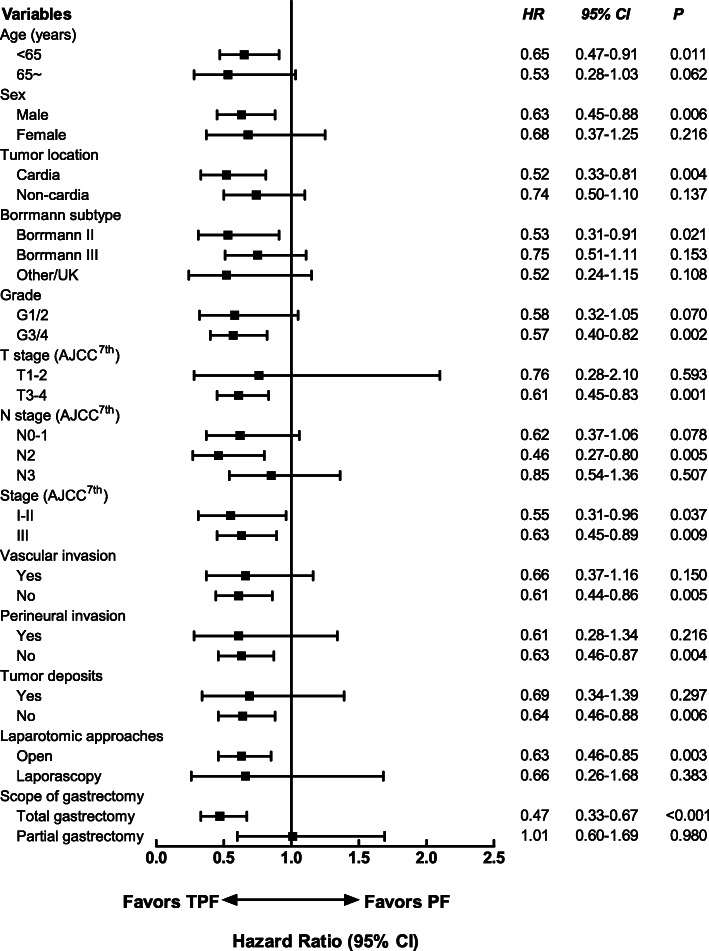
Forest plot of hazard ratios and 95% confidence intervals for disease-free survival by characteristics of patients. PF, platinum plus fluoropyrimidines; TPF, taxanes, platinum plus fluoropyrimidines; AJCC, American Joint Committee on Cancer; HR, hazard ratio; 95% CI, 95% confidence interval

**Fig. 5:**
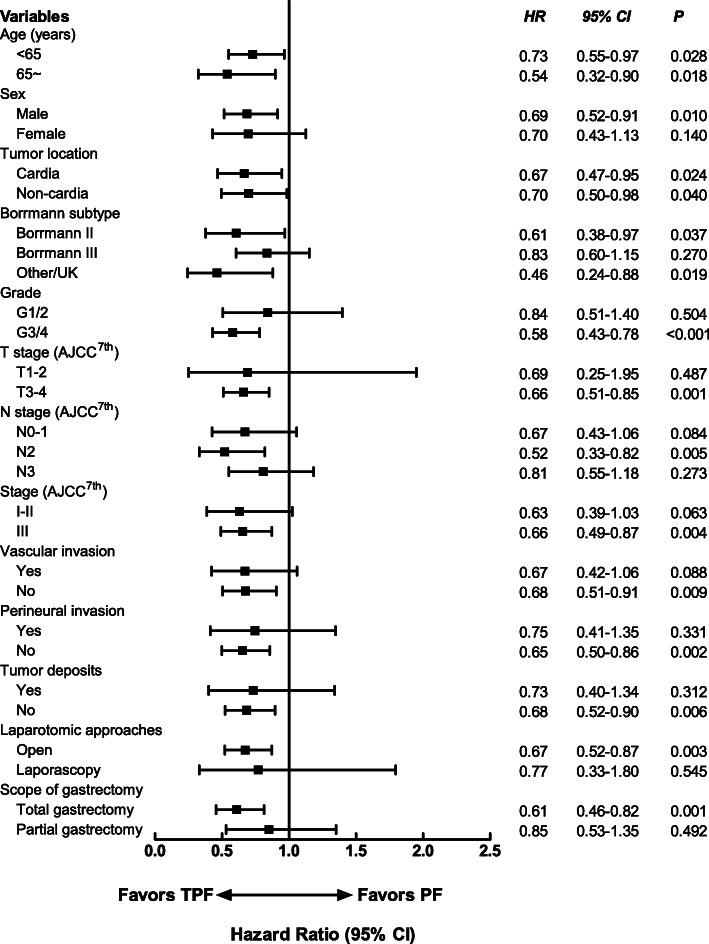
Forest plot of hazard ratios and 95% confidence intervals for overall survival by characteristics of patients. PF, platinum plus fluoropyrimidines; TPF, taxanes, platinum plus fluoropyrimidines; AJCC, American Joint Committee on Cancer; HR, hazard ratio; 95% CI, 95% confidence interval

Figures 4 and 5 showed forest plot of hazard ratios for DFS and OS by different characteristics. The risk of disease recurrence as well as death was reduced in patients less than 65 years receiving TPF versus PF (HRs were 0.65 [*95% CI*, 0.47~0.91] and 0.73 [*95% CI*, 0.55~0.97], *P* values were 0.011 and 0.028, respectively). For male patients and those with cardia cancer, Borrmann II subtype tumors, G3-4 tumors, T3-4 tumors, N2 tumors, stage III disease, without vascular or perineural invasion, without tumor deposits, and patients who receive open laparotomy and total gastrectomy, the risk of recurrence and death were also lower. The risk of death was also reduced in elder patients (65 years old and more; *HR* = 0.54; *95% CI*, 0.32~0.90, *P* = 0.018) and patients with non-cardia tumors (*HR* = 0.70; *95% CI*, 0.50~0.98; *P* = 0.04) who receiving TPF regimens, but no significant differences in PFS were observed with TPF versus PF in these patients.

## Discussion

In Asian countries, the survival of gastric cancer after D2 gastrectomy was further improved by adjuvant capecitabine plus oxaliplatin or adjuvant S-1 which demonstrated in the CLASSIC trial and ACTS-GC trial [4, 6]. Adjuvant fluoropyrimidine monotherapy or in combination with platinum was also considered as standard of care for resectable gastric cancer. The addition of docetaxel or paclitaxel in chemotherapy regimens was shown to be effective in advanced gastric cancer [13–15] as well as in preoperative or neoadjuvant settings [16, 17]. Intensive chemotherapeutic regimens with triple-drug combination were seldom tested in adjuvant settings. In the CALGB 80101 study, after curative resection of gastric or gastroesophageal junction adenocarcinoma, adjuvant chemoradiotherapy using the three-drug regimen of epirubicin, cisplatin, and infusional FU (ECF) did not improve survival compared with chemoradiotherapy with bolus fluorouracil and leucovorin [18]. By using PSM analysis, present retrospective study demonstrated that the addition of docetaxel or paclitaxel to the conventional combination of platinum and fluoropyrimidines in adjuvant chemotherapy for patients after curative gastrectomy could prolong disease-free survival and overall survival. As far as we know, triplet regimens as postoperative adjuvant therapy has not been tested in clinical trials, our results offered optional regimens for gastric cancer patients who received D2 gastrectomy.

As we known, both of paclitaxel and docetaxel were demonstrated as effective drugs in gastric cancer. While introducing taxanes into adjuvant regimens, randomized controlled JACCRO GC-07 trial demonstrated the superiority of S-1 plus docetaxel to S-1 for 3-year relapse-free survival (66% vs. 50%) in patients with stage III gastric cancer [9]. But sequential administration of paclitaxel followed by fluoropyrimidines did not improve DFS compared to tegafur and uracil (UFT) or S-1 monotherapy [8]. Recently, adjuvant albumin-bound paclitaxel was also planned to be tested in Chinese gastric cancer patients [19].

While comparing to results in CLASSIC and ACTS-GC trial, the absolute survival times were shorter in our study. We attributed these to the following reasons: (1) patients enrolled in clinical trials were usually of better performance status than patients in the real clinical practice, with well tolerance and compliance; (2) in CLASSIC and ACTS-GC trial, stage II disease accounted for about 50% of enrolled patients [3, 5]; however, in our study, more than 60% were stage III disease; (3) due to retrospective design of our study, we selected cases merely based on the stored files and medical records, biases were inevitably existed in the description of surgical procedures, adjuvant chemotherapy, and follow-up details; (4) some important information cannot be obtained in our study, for example, host’s performance status, immune-inflammatory status, comorbidity, and postoperative complications, these factors were considered to have an influence on the survival of the patients [20–25]. However, as we know, for patients with malnutrition and (or) anemia, the performance status is usually poor; on the other hand, for patients after gastrectomy, if severe postoperative complication occurred, the incidence of anemia and hypoproteinemia would be high. So, to some extent, the concentration of hemoglobin and level of albumin can be as surrogates of performance status and complications.

Finally, due to the limitations of data acquisition and the retrospective design, treatment-related toxicity could not be fully retrieved in our study, so we did not list the differences of side effects between two groups. However, it can be postulated that, patients undergoing intensive chemotherapy may experience greater toxicities, which has been observed in clinic trials for advanced gastric cancer [13, 14].

## Conclusions

We concluded that adjuvant chemotherapy with taxanes, platinum, and fluoropyrimidines for gastric adenocarcinoma after curative gastrectomy showed survival benefit compared to doublet regimen of platinum and fluoropyrimidines. Further prospective randomized trials are encouraged to conduct and to verify the effectiveness of taxanes in the adjuvant setting.

## Data Availability

The data that support the findings of this study are available from the corresponding author on reasonable request.

## Abbreviations

SAMIT: Stomach Cancer Adjuvant Multi-Institutional Group Trial
AJCC: American Joint Committee on Cancer
HIS: Hospital Information System
PSM: Propensity score matching
DFS: Disease-free survival
OS: Overall survival
HR: Hazard ratio
CIs: Confidence intervals
PF: Platinum plus fluoropyrimidines
TPF: Taxanes, platinum, and fluoropyrimidines
CLASSIC: Capecitabine and Oxaliplatin Adjuvant Study in Stomach Cancer
ACTS-GC: Adjuvant Chemotherapy Trial of S-1 for Gastric Cancer
CALGB: Cancer and Leukemia Group B
JACCRO: Japan Clinical Cancer Research Organization
